# Dietary Energy Density and Its Association with Overweight or Obesity in Adolescents: A Systematic Review of Observational Studies

**DOI:** 10.3390/nu10111612

**Published:** 2018-11-01

**Authors:** Andrea Arango-Angarita, Sonia Rodríguez-Ramírez, Lluis Serra-Majem, Teresa Shamah-Levy

**Affiliations:** 1 Center for Evaluation and Surveys Research, National Institute of Public Health, Cuernavaca, 62100 Morelos, Mexico; andreaarango20@gmail.com; 2 Center for Nutrition and Health Research, National Institute of Public Health, Cuernavaca, 62100 Morelos, Mexico; scrodrig@insp.mx; 3 Research Institute of Biomedical and Health Sciences, University of Las Palmas de Gran Canaria, 35016 Las Palmas de Gran Canaria, CP, Spain; lluis.serra@ulpgc.es

**Keywords:** energy density, dietary energy density, overweight or obesity, weight gain, adolescents

## Abstract

Dietary energy density (DED) has been identified as a crucial dietary factor in body weight control, in that higher DED has been associated with weight gain. To our knowledge, no review studies have explained this association specifically in adolescents. The aim of this study was to describe the association of DED with overweight or obesity (OW/O) in adolescents, as derived from observational studies. We conducted a systematic search of the MEDLINE/PubMed and Science Direct databases, including studies published between January 2000 and December 2017. We selected the studies that included adolescents (aged 10–19 years) and contained DED-related information and anthropometric measurements of OW/O. From 1149 candidate studies, 30 were selected, though only 12 met all the inclusion criteria. Of these, only four found a positive association between DED and certain OW/O indicators, six found no association and two showed an inverse association with weight gain. These studies differed in several aspects such as design, DED calculation method and dietary assessment tool, leading to inconsistent results. Methodological differences found among the examined studies did not allow us to establish a clear conclusion of this association. Evidence in adolescents was also poor. New, standardized methodological approaches should be considered in future studies.

## 1. Introduction

Adolescence is a critical period of life characterized by physical, physiological, cognitive, social and emotional changes that could affect food consumption and food habits [1,2]. Many factors influence adolescents’ eating behaviors: cultural beliefs, mass media, body image, food preferences and others [1]. Adolescents usually include energy-dense products in their diet [2], skip meal times, and follow unstructured and out-of-home eating patterns that could be related with obesity and weight gain [3,4].

Overweight and obesity (OW/O) are multifactorial diseases characterized by an excess of adipose tissue resulting from extra energy intake in relation to total energy expenditure [5]. The dramatic worldwide increase in the prevalence of OW/O in adolescents is considered a serious public health issue because of its direct association with changes in health and quality of life starting in the early stages of life [5,6]. From 1980 to 2013, the combined prevalence of OW/O in developed countries increased from 8.25 to 13.5% in children and adolescents aged 2–19 years, and about 23.2% of the child population in developing countries presented this condition in 2013 [7]. Overweight or obese children and adolescents are more likely to have obesity in adulthood, as well as have psychological and social difficulties, and chronic degenerative diseases such as heart disease, cancer and type 2 diabetes [5,8].

Some dietary factors, such as dietary patterns based on the Mediterranean diet, have been widely associated with reduced OW/O and their comorbidities [9], though others have been associated with weight gain [10]. Recent research focused on the study of dietary energy density (DED) has clarified its causal involvement with developing OW/O, as well as its role in changes in body weight [10,11] and as a possible indicator of diet quality [12,13]. 

DED is defined as the amount of energy available in a given food weight [14,15], generally expressed in kcal/g or kJ/g [15]. DED can be calculated either excluding or including beverages [16]. DED depends on the fiber, water and fat content in the diet [15]. Fiber and water provide weight to food, fewer calories, and a greater sense of satiety, making foods rich in these components have a lower DED, provide higher nutrient density, and improve diet quality [12,15]. In contrast, high-fat foods generally have higher energy density because the energy content of fat (9 kcal/g or 38 kJ/g) is higher than that of other macronutrients, with considerably increased DED [15]. This is especially likely if it is accompanied by sugar, as is the case with some processed or ultra-processed foods, which have also been associated with weight gain in adolescents [17]. 

Previous systematic reviews [10,16] and meta-analyses [11] have documented that high-energy diets are a risk factor for weight gain. An association has been found between DED and increased body mass index (BMI) [18] and/or waist circumference (WC) [11,18] in adults, where associations with metabolic syndrome have also been reported [19]. In children, evidence of the association of DED with OW/O is weaker [11,20,21] and is related with the ability [22,23] or inability [24,25] to compensate for energy intake. However, some observational studies have documented that high DED intake leads to increased adiposity [20,26] and weight gain [27]. In adolescents, the evidence is inconsistent [28]. Additionally, there are no current studies or reviews that provide a clear conclusion regarding the association of DED with weight gain or OW/O in this age group. Therefore, the aim of this systematic review is to describe the association of DED with OW/O in adolescents, as derived from observational studies.

## 2. Methods 

### 2.1. Search Strategy

We conducted a systematic review of original, English-language articles from observational (cohort and cross-sectional) studies that analyzed the association of DED with weight gain and/or OW/O in adolescents, and that were published between January 2000 and December 2017. We used the MEDLINE/PubMed and ScienceDirect databases. Medical Subject Headings (MeSH) and keywords were used to perform an advanced search for articles in MEDLINE/PubMed. “Diet or dietary”, “energy or energy intake”, “density”, “overweight or obesity” and “adolescents or adolescence” were used as keywords or MeSH terms. For ScienceDirect, we searched journals for all fields using “dietary energy density or energy density” and “overweight or obesity” and “adolescents, adolescence or young”. Some of the articles were selected manually, based on references in previously reviewed articles.

The search was conducted three times—in May 2016, August 2017 and January 2018—to ensure that all possible eligible studies were addressed. The search results were imported into Mendeley software and the citations were downloaded for review in text (txt file) format. The present study followed the PRISMA Statement for systematic reviews [29].

### 2.2. Eligibility Criteria

#### 2.2.1. Studies

Prospective (cohort) and cross-sectional studies published worldwide between 2000 and 2017 were selected. We included studies with a sample size of >30 individuals per study. Duplicate studies were excluded, as were reviews, clinical trials, meta-analyses, letters to the editor, and editorials.

#### 2.2.2. Subjects 

We considered studies with adolescents aged 10–19 years (for some cohort studies, we considered age at follow-up but not age at the beginning of the study), healthy and/or diagnosed as overweight or obese. However, studies that included hospitalized subjects, those undergoing medical treatment, those with another disease such as cancer, those under treatment for weight loss or malnutrition, as well as lactating or pregnant women, were excluded.

#### 2.2.3. Variables 

DED was considered as the exposure, as well as the main variable of diet; conversely, OW/O was defined as the outcome. Additionally, we included studies that considered anthropometric measures such as BMI, BMI z-score, % body weight, WC, fat mass index (FMI) or body fat percentage (%BF) as an indicator of OW/O. There was no restriction on the selection of studies by dietary collection instrument.

### 2.3. Study Selection

After the initial review of databases, a txt file was used to manually identify duplicate studies. Then, selection was made using the following steps: Duplicate studies were removed using Mendeley. Potentially eligible studies were then selected by reading the title and/or abstract. After that, the complete text of selected studies was read and relevant information was extracted into tables, selecting only studies that met the inclusion criteria. Finally, references of the selected studies were reviewed, including those not found in the databases but mentioned in those studies. Likewise, other systematic reviews were reviewed to consider all related studies.

The quality of the cross-sectional and cohort studies included in the review was assessed using the Newcastle-Ottawa Scale. A nine-point scoring system for cohort studies and 10-point system for cross-sectional studies were used. In this tool, each study is appraised on eight items, categorized into three groups: selection of study groups, comparability of groups, and ascertainment of either the exposure or outcome of interest for cohort studies [30]. For cross-sectional studies, an adapted Newcastle–Ottawa Scale was used [30]. A high-quality study was defined as having a score equivalent to ≥7 points for cohort and ≥8 for cross-sectional studies.

### 2.4. Data Extraction

A data extraction form was made including the following information: author, year of publication, study design (prospective or cross-sectional); location of study, number of participants (recruited, eligible, enrolled); age at which reference and follow-up measures were performed, diet assessment tool, DED calculation method, OW/O measurement indicator, the final Newcastle–Ottawa score, statistical analysis methods, DED variable type (continuous or categorical); and other measurements such as underreporting and potentially confusing variables, and finally the results or conclusions of the study.

## 3. Results

**Figure 1:**
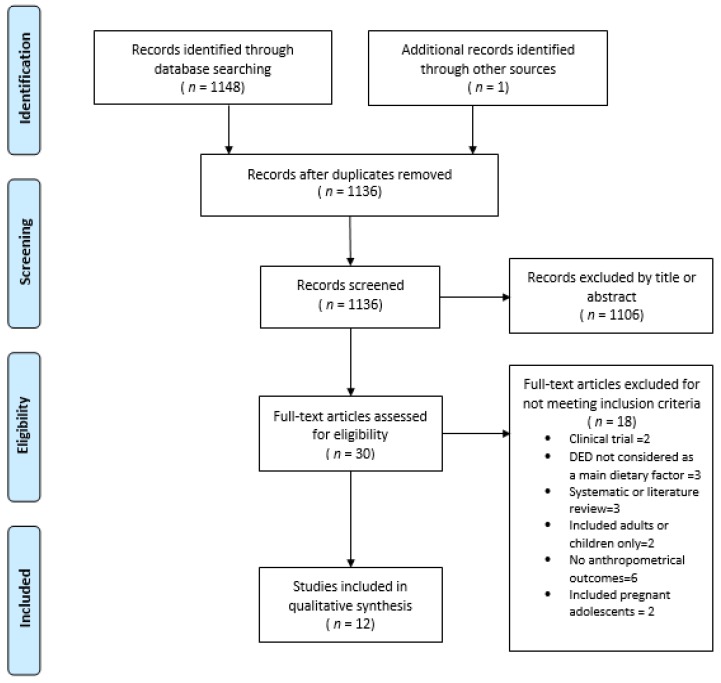
Flow chart illustrating the study selection process.

This review was based on studies that analyzed the association between DED and OW/O in adolescents through indicators such as weight gain, BMI, BMI z-score, fat mass, %BF and FMI. After applying the search filters by MeSH terms, keywords or all fields, and removing duplicate studies, 1148 studies were reviewed: 165 from MEDLINE/PubMed and 983 from ScienceDirect. From MEDLINE/PubMed, 21 studies were selected by title and abstract, of which nine fulfilled the inclusion criteria (full text reading). Eight were selected from ScienceDirect, but only two (non-replicated studies) were included. One study was identified manually. Twelve articles were included in the present review (Figure 1).

### 3.1. Characteristics of Studies 

Table 1 shows the characteristics of the included studies. Eight cohort studies [28,31,32,33,34,35,36,37] were carried out in the United States, Germany, United Kingdom, Ireland, Denmark and England, and four with cross-sectional design [38,39,40,41] were carried out in Japan, China, Australia and Spain. All selected studies were published between 2004 and 2016, in adolescents aged 10–19 or children aged 6–11 years who were followed up until adolescence [28,32,33,35]. The sample size range was 40–8202children and/or adolescents; five studies had <500 subjects [28,31,32,33,35], three had 500–2000 participants [37,39,40] and four had >2000 [34,36,38,41] (Table 1).

As per the Newcastle–Ottawa scale, all the selected studies showed high-quality methodology (≥7 points in cohort studies or ≥8 points in cross-sectional studies). 

#### 3.1.1. Anthropometric Measurement

For measurement of OW/O, most studies used indicators such as BMI z-score [35], BMI [38], standard deviation scores of BMI, [31] WC, [41], body weight [37], FMI [34,36] or considered two of these indicators such as the BMI [40] or BMI z-score [33] and FMI [33] or waist–height ratio (WHR) [40]. Three studies performed measurements with more than four of the indicators mentioned above, including BMI z-score [32,39], FMI [29,32,39], %BF [28,32,39], fat mass (FM) [32], fat free mass (FFM) [32], fat free mass index (FFMI) [39], WC [28,32], body weight [32], and/or WHR [39] (Table 1).

#### 3.1.2. Diet Instrument

Four studies measured diet with a 24-h dietary recall (24 HR) [35,37,39,41], six by dietary record (three with weighed food record) [28,31,32,33,34,36], one used a food frequency questionnaire [40] and another used a dietary history questionnaire [38] (Table 1).

#### 3.1.3. DED Calculation Method 

Four studies included solid food only [28,36,38,41], three included solids and beverages [31,34,35], one excluded only water or beverages without energy [37], and four used three or more methods of calculating DED, which included solid foods only, solid foods and beverages, solid food and milk, solid and high-calorie beverages and others [32,33,39,40] (Table 1). 

On the other hand, DED was mainly expressed as a continuous variable [28,31,32,35,36,37,40] or as categorical variable in tertiles [33,39], quartiles [41] and quintiles [38] of DED or using the DED calculation method [32]. The range of DED was 1.32–8.64 kJ/g and DED was higher for those that included solid foods only and lower for those that included solid foods and beverages (Table 2).

### 3.2. Analysis of the Association between DED and OW/O

Most studies determined the association of DED with certain OW/O measures through β coefficients [33,34,35,36,37], odds ratios [32,38] or both [28,40,41], as well as clusters or conglomerates [31] and linear trends [39]. The main adjustment variables of the models were sex, age, maternal educational level, energy (underreporting), physical activity, screen time, puberty (Tanner stage or from which a sum-score (0–5) was derived from three general scores (growth spurt, body hair growth, spots/acne) and two sex-specific scores) [28], parental overweight, energy from sugar-sweetened beverages, energy intake and fiber intake. In six studies, energy underreporting was calculated using the ratio between energy intake (EI) and estimated energy requirement (EER) (EI:EER) [28,34,35,36,38,40]. Two studies presented the ratio of energy intake/energy expenditure (EI:EE) which was determined using doubly labeled water [32,33] and two using Goldberg cut-off points (EI:BMR) [31,41] using predictive formulas to determine basal metabolic rate (Table 2).

### 3.3. Characteristics of Studies with Positive Associations between DED and OW/O

Four studies reported a positive association between DED and certain OW/O indicators such as FMI [32,34,36] and WC [41]. In contrast, five did not find any association between DED and any OW/O measure [33,35,37,38,39], one found association with WC at baseline but not at follow-up [28], and two identified an inverse association [31,40].

Studies that found a positive association between DED and OW/O used dietary records (weighed food records) [32,34,36] or 24 HR [41] for assessing diet and excluded all beverages [36,41] or included solid foods and beverages [34] as the DED calculation method. In a study, it was performed using five different methods of calculating DED: ED all—all foods and all energy-containing beverages and energy-free beverages, including water; ED foods—all foods, milk as food; ED soup—all foods, milk as food, and soups; ED solid—all solid foods; and ED energy—all foods, milk as food, soups and energy-containing beverages with a caloric density greater than 21 (kJ/100 g). However, associations with FMI were found only with methods that excluded all beverages [32]. The mean DED reported in the studies was 6.2 kJ/g [41], 8.64 kJ/g [36] and 8.22 kJ/g (ED soup), 8.28 kJ/g (ED food) and 9.17 kJ/g (ED solid) [32].

Four studies found association of DED with FMI, showing β coefficients between 0.03 (95% confidence interval (CI) (0.01–0.03) and 0.16 ± 0.06 and with WC in 0.72 (95% CI 0.26–1.17) [28] and 1.922 (95% CI 0.974, 2.510) [41]. Conversely, studies that found no association between DED and BMI z-score, such as Kring et al. [35], presented coefficients of −0.04 (95% CI −0.29, 0.20) in boys and 0.23 (95% CI −0.07, 0.53) in girls, and Butte et al. [37], in which the coefficients were between 0.23 ± 0.35 and 0.24 ± 0.39 with different models, but did not show significant association with weight gain.

However, odds that indicated a positive association of DED with OW/O were found between 2.164 (95% CI 1.099, 4.251) and 1.940 (95% CI 1.054, 3.571) in McCaffrey et al. [32], with slight variations based on the calculation method used. In contrast, studies that did not find associations with any OW/O indicator, such as Murakami et al. [38], showed odds of 0.78 (95% CI 0.57, 1.07) in boys and 0.85 (95% CI 0.61–1.20) in girls, on the last quintile of DED. O’Sullivan et al. [40] showed odds of 0.83 (95% CI 0.70, 0.99) and 0.87 (95% CI 0.77, 0.99) and Van Sluijs et al. [28] presented odds at study follow-up of 1.02 (95% CI 0.83, 1.26) and 0.94 (95% CI 0.71, 1.23) (Models 1 and 2, respectively) (Table 2).

## 4. Discussion

This review included 12 studies that analyzed the association between DED and OW/O, but only four found a positive association between DED with some indicator of adiposity. The results of the association of DED with OW/O in adolescents provided in our review were inconclusive and the evidence found was limited. In agreement with our findings, Perez et al. [10] and Rouhani et al. [11] found wide differences in the design and methodology from the reviewed studies. According to the Dietary Guidelines for Americans 2010 (DGAC), there is enough evidence from longitudinal studies to support the association between DED and OW/O [10]; however, the evidence is more consistent for adults than for children or adolescents, since a large number of studies in adults (from both cohort and clinical trials), has shown clear results, mainly in the association between low DED and weight loss, under high-quality methodological criteria [10,11]. Differences seem to be related with the response to DED effects and to variations in energy density between age groups [15]. Additionally, dissimilarities in estimations of DED (related to specific dietary reporting patterns in adolescents) [42] and OW/O between adolescents and adults, could also influence the strength of the results in each population. Despite this, the recommendation to reduce DED and increase the consumption of foods with low energy density remains a key strategy for the prevention of OW/O in all age groups.

Differences observed between studies seem to be the result of methodological factors that could have attenuated or modified the associations of DED with OW/O in adolescents. First, most studies were longitudinal, which allowed associations to be found because of the causal inference capacity. However, some studies showed positive associations at the study outset [28], but not at follow-up, in the course of puberty [33,35] or in adolescence [28,37]. Other studies have shown that the clearest association between DED and adiposity in young people occurs when sample is restricted to children (<10 years) [20,26]. This suggests that the mechanisms by which DED acts in adolescence differ from those observed during childhood, where the response to variations in energy density seems to occur innately [11,22]. However, it has been documented that increased energy density leads to higher energy intake in children [24] and that the ability to compensate for dietary energy intake decreases progressively with age [14,26]; thus, the probability of weight gain with a high DED intake should be higher during adolescence. This contrasts with our results, in which the DED response and associations with weight gain was practically unclear.

On the other hand, studies that found an association of DED with a certain OW/O indicator excluded all beverages [36,41] or performed different combined methods for DED calculation [32]. This agrees with other studies in children and adults [44,45] that found positive associations using this calculation method. It is well known that beverage consumption gives a weaker and differential effect in control and regulation of appetite and satiety, and its inclusion in DED calculation could alter interpretation of the results [16,46]. This could have limited comparability between studies [47], whereby the evidence has recommended independently evaluating the DED of solid food and beverages [46] or excluding beverages in the DED calculation but including its calories as a covariate [16]. Despite this, Zhou et al. [39], O’Sullivan et al. [40], Gunther et al. [33] and Van Sluijs et al. [28] did not find any association, even when using four different methods to calculate DED or adjusting the models with energy from beverages.

Most studies included BMI [31,38,40] or BMI z-score [35] as an anthropometric indicator of OW/O in the population; however, the studies that found association between DED and OW/O in adolescents used FMI as a predictor of adiposity [34,36]. Although some other studies found no association, even when using FMI as an indicator of fat mass measurement [28,39], they did not use techniques such as dual energy X-ray absorptiometry (DXA) [34,36] or doubly marked water to estimate energy expenditure [32] to obtain unbiased information, which may have improved the results. A recent study found that the tri-ponderal mass index (TMI) (mass divided by height cubed) estimates body fat levels more accurately than BMI z-score in adolescents, which may be a superior alternative to improve the likelihood of associating DED with excess body weight [48]

In relation to dietary assessment, dietary record (by weighing) and 24 HR were commonly used in studies that found an association between DED and adiposity [32,34,36,41]. Although the biases inherent in the instrument could result in underreporting and/or over-reporting energy intake (mainly in 24 HR), both instruments have been described as the most appropriate methods for accurately estimating absolute energy intake [49]. According to Burrows et al. (2010) [50], dietary history provides better estimates of food intake in adolescents compared with other instruments such as dietary record and 24 HR (3 days a week). However, most studies found misreporting as one of the main causes of error in dietary assessment [28,37,38], mainly due to underreporting, which occurs frequently in adolescents [42,49,51]. In this age range, underreporting generally provides lower intakes of snacks and energy-dense foods than plausible reports [51] because adolescents tend to easily forget and/or omit foods they consume [51]. Moreover, it has been documented that the exclusion of non-plausible reporters can improve the magnitude of dietary (including DED) associations with obesity [52,53]. Likewise, some studies have indicated that associations tend to be stronger when under-reporters are excluded from analysis or this variable is introduced as a covariate [52,53,54].

The World Cancer Research Fund recommends average DED to be reduced to <5.3 kJ/g or <525 kJ/100 g and considers energy-dense foods as those with an energy content of more than about 9.4–11.5 kJ/g or 941.1–1150.6 kJ/100 g) [43]. Considering the above, studies that reported a lower mean DED—as 3.9 kJ/g [31], 4.46 kJ/g [40] or 5.1 kJ/g [38]—found no association with weight gain in adolescents. In contrast, studies that reported mean DED of 8.64 kJ/g [36] and 9.17 kJ/g [32] (excluding all beverages), found positive results. However, some studies, such as Zhou et al. (6.5 kJ/g) [39] and Van Sluijs et al. (7.7 kJ/g at follow-up) [28], obtained relatively high mean DED and found no association with any OW/O indicator.

Regarding adjustment variables, many studies adjusted for variables known to be predictors of obesity [55], but these did not identify any type of association between DED and OW/O. However, inaccuracies in the measurement of variables such as physical activity, screen time, and puberty probably increased the inconsistencies observed in the studies; for example, Ambrosini et al. [34] provided evidence that physical activity and puberty acted as negative predictors of weight gain while, in contrast, educational level and pre-gestational BMI increased association with FMI. However, Butte et al. [37] identified maternal BMI, birth weight, early sexual maturation, screen time, and sleep time as significant predictors of weight gain, though not other variables. Moreover, some OW/O risk factors, such as alcohol consumption and smoking, were not considered within the reviewed studies. However, considering they are variables of special importance in adolescence, they could be considered as adjustment variables in models.

### Limitations

There are some limitations in this review. First, the differences between studies did not allow quantitative analysis (meta-analysis) that would have estimated the pooled effect size. Second, it is possible that the search strategy used in the selected databases did not detect all eligible studies, especially in ScienceDirect, where a large number of studies did not correspond to the main topic searched. However, the repeated reviews made by advanced search in MEDLINE/PubMed and the revision of references in other studies could have reduced this limitation. Third, the majority of the examined studies were conducted in Europe and in developed countries, which does not allow generalization of the results to all populations, as these could vary considering the different characteristics and social conditions of each country. A strength of this review is that it synthesizes information about DED and OW/O in adolescents, based on high-quality studies, providing useful information and methodological approaches that could lead the way to novel research.

## 5. Conclusions

The differences in study designs, as well as variability in DED calculation method, the dietary assessment tools used, the misreporting of estimates, and the age of included groups, made it impossible to clarify the magnitude of the association between DED and OW/O in adolescents. Further research is therefore needed. We recommend that future studies develop and implement new methods of collecting information, including new technologies for assessing dietary intake, OW/O measurements other than BMI (e.g., FMI or TMI) and including only solid food from the DED calculation method. When analyzing information, it is necessary to exclude implausible dietary energy reporters and/or to adjust for underreporting (with accurate estimates of total EI and EER), as well as for energy intake from beverages and OW/O predictor variables typical of adolescence.

## Figures and Tables

**Table 1:** Characteristics of the eligible studies reviewed.

Author	Country/Study	Design	Years Follow-Up	Age and N Subjects	Diet Instrument	DED Calculation Method	Measurement Indicator OW/O	New-Castle Ottawa Scale ^1^
Alexy et al. (2004) [31]	Germany/DONALD	Cohort	2	228 boys and girls 2 to 18 years	Dietary record	Foods and beverages	Mean of SDS of BMI ^2^	8
Ambrosini et al. (2012) [34]	England/ALSPAC Avon Study	Cohort	8	6672 boys and girls 7 to 15 years	Dietary record, 3-day unweighed food diary	Foods and beverages	FMI^3^, Risk of obesity	8
Butte et al. (2007) [37]	United States/, Viva la Familia Study	Cohort	1	879 boys and girls 4 to 19 years	24-h dietary recall	Includes food and energy containing beverages and excludes non–energy containing beverages and water	Weight gain ^4^ (kg/years)	7
Gunther et al. (2011) [33]	Germany/DONALD	Cohort	3	219 boys and girls Mean 6.9 years at baseline 9.4 y ATO	Dietary record	ED_all: included all foods and drinks ED_energy: included all foods and energy containing drinks (5 kcal/100 g). ED_milk: included all foods and milk as a drink, but no other beverages. ED_food: included foods only (solid/liquid).	BMI z-score, FMI ^5^	7
Johnson et al. (2009) [36]	England/Avon Study	Cohort	3	2275 boys and girls 10 to 13 years	3-day unweighed diet diaries	Solid food only	Fat mass, FMI ^3^	8
Kring and Heitman (2008) [35]	Denmark	Cohort	3	398 boys and girls 8 to 10 years 9.6 years at baseline	24-h dietary recall	Foods and beverages	BMI Z-Score	8
McCaffrey et al. (2008) [32]	Ireland	Cohort	8	48 Boys/girls; 6–8 years at baseline and followed up at 13 to 18 years	7-d weighed food records	ED all: All foods and all energy containing beverages and energy-free beverages and water. ED Foods: All foods, milk as food. ED soup: All foods, milk as food, and soups. ED Solid: All solid foods ED Energy: All foods, milk as food, soups, and energy-containing beverages	Body weight, BMI z-score, WC, FFM ^6^, FMI ^6^, %BF ^6^	7
Murakami et al. (2012) [38]	Japan/RYUCHS	Cross-sectional	NA	15,974 children and 8202 adolescents. Children 6 to 11 years. Adolescents 12 to 15 years	Diet history questionnaire BDHQCA	Solid food only	BMI ^7^	8
O’Sullivan et al. (2015) [40]	Australian	Cross-sectional	NA	1613 boys and girls 14 years	FFQ	Food only, food and all beverages, food and all beverages excluding water, and food and energy containing beverages	BMI ^8^ and WR	8
Schröder et al. (2013) [41]	Spain	Cross-sectional	NA	2513 adolescents 10 to 24 years	24-h dietary recall	Solid food only	WC	8
Van Sluijs et al. (2016) [28]	United Kingdom/SPEEDY Study	Cohort	4	367 adolescents 10 years at baseline, 14 years at follow-up	4-d food diary	Solid food only	WC, % BF ^9^, FMI^9^, weight status	9
Zhou et al. (2015) [39]	China	Cross-sectional	NA	1207 boys and girls 8 to 14 years	24 -h dietary recall	ED1: Foods and beverages ED2: Solid food only ED3: All solid foods and milk ED4: solid foods and energy-containing beverages (>21 kJ or 5 kcal per 100 g) ED 5 Included solid foods, and milk and energy-containing beverages	BMI z-score, % BF^10^, FMI ^10^, FFMI ^10^ and WHR	8

^1^ Newcastle–Ottawa Scale was used only to score the quality of studies; ^2^ BMI was converted into standard deviation scores of BMI; ^3^ FM was measured by dual-energy X-ray absorptiometry (DXA) and FMI: FM/height^2^. ^4^ BMI was used and body composition was determined by dual-energy X-ray absorptiometry; ^5^ %BF was estimated using Slaughter equations for pre-puberty and puberty (ATO) and converted to FMI Z-scores; ^6^ %BF was calculated as body fat (in kg)/body weight (in kg) × 100; Fat-free mass (FFM) was calculated from total body water by dividing the water content of fat-free tissue with age- and sex-specific values, Fat mass (FM) was calculated as the difference between body weight and FFM then FMI was expressed as FMI: FM/height^2^; ^7^ BMI was calculated using International Obesity Task Force age—and sex-specific BMI cutoffs for children; ^8^ BMI was calculated using standard criteria for adolescents; ^9^ Previously validated procedures using eight equations were used to calculate fat mass and %BF [28]; FMI: FM/height^2^; ^10^ %BF was calculated using the Slaughter equations [39]; FMI was calculated using ((weight × %BF)/height^2^) formulae; FFMI was calculated as: ((weight − (weight × %BF))/height^2^). Abbreviations: ATO: age at take-off; ALSPAC: Avon longitudinal Study of Parents and Children; BDHQCA: Diet history questionnaire for Japanese children and adolescents; %BF: body fat percentage; BMI: Body mass index; DED: Dietary energy density; DONALD: Dortmund Nutritional Anthropometric Longitudinally Designed Study; ED: energy density; FFM: Fat free mass; FFMI: Fat free mass index; FMI: Fat mass index; FFQ: food-frequency questionnaire; NA: does not apply; OW/O: Overweight or obesity; RYUCHS: Ryukyus Child Health Study; SDS: Standard deviation score; SPEEDY: Sport, Physical activity and Eating behavior: Environmental Determinants in Young people; WC: Waist circumference; WHR: waist-to-hip ratio; WR: Waist height ratio.

**Table 2:** Characteristics and results of the eligible studies reviewed.

Author	Type of Variable (DED)	Statistical Analysis	Energy (Underreporting) Estimate	Adjustment Variables	DED Value/Mean (kJ/g/kcal/g) ^1^	β, OR, *p* Value	Results
Alexy et al. (2004) [31]	Continuous	Cluster analysis	EI:BMR Goldberg formula to estimate plausibility of energy intake	Sex, age, education level, occupation level of parents, anthropometric characteristics (BMI), energy, macronutrient and food group intakes.	3.9	Cluster of fat intake pattern DED *p* < 0.0001 Medium 4.0 (0.4) High 4.1 (0.4) Low 3.7 (0.4) BMI *p* 0.05 Medium 0.11 (0.85) High 0.06 (0.88) Low 0.26 (0.70)	During the study period, the highest SDS of BMI was observed in the low fat cluster (*p* 0.05) and the DED was lowest in the low fat cluster (*p* < 0.001)
Ambrosini et al. (2012) [34]	Categorical: Quintiles of DP at 7, 10 and 13 year	Multiple linear regression model	EI: EER Individuals were categorized as plausible, underreporters and overreporters. It was included as a categorical covariate in all analyses	Model 1: Age, sex and dietary misreporting Model 2: model 1 + physical activity Model 3: model 1 + maternal education level and maternal pre-pregnancy BMI	NR	13 years quintile 1 and 2 Model. β 0.03 95% CI (0.01–0.03) *p* 0.003. 3. Model. β 0.01 95% CI (0.01–0.03) *p* 0.348	Energy-dense, high-fat, low-fiber dietary patterns are positively associated with a higher FMI.
Butte et al. (2007) [37]	Continuous	GEE population-averaged panel data models, multiple lineal regression	NR	Model 1: Age, sex, age squared, Tanner stage Model 2:Model 1 + BMI status	1.32	Model 1.β 0.24 ± 0.39 *p* 0.53 Model 2. β 0.23 ± 0.35 *p* 0.50	No significant association was found between DED and weight gain.
Gunther et al. (2011) [33]	Categorical: Tertiles of ED (T1–T3)	Multiple linear regression model	NR	Sex; birth year; birth weight, maternal overweight, maternal age at birth, protein percentage of total energy intake, fat, fibre and baseline BMI/FMI Z-score.	ED all 4·1 ED energy 5·1 ED milk 6·0 ED food 6·9	Lesmean (Least square mean) BMI Z-score at ATO Tertile l. 0 CI (−0.1, 0.2) Tertile 2. 0 CI (−0.1, 0·1) Tertile 3. 0 CI (−0.1, 0·2) *p* 0·8 FMI Z-score at ATO Tertile l. 0 CI (−0.2, 0.1) Tertile 2. −0.1 CI (−0.2, 0·1) Tertile 3. 0.1 CI (−0.1, 0·2) *p* 0·9	DED was not associated with BMI z score and FMI at age of pubertal takeoff. DED in childhood did not influence timing or body fatness at ATO.
Johnson et al. (2009) [36]	Continuous	Multivariate models, multiple linear regression model	EI:EER Categorical misreporting variable (under-, plausible-, and over-reporter) was used as a covariate in regression analyses	Model 1: DED, sex, height at age 13 years, misreporting of energy intake Model 2: Model 1 + Puberty, overweight status at 10 years, energy intake of drinks, maternal education, TV watching, and physical activity	8.64	DED β 0.21 ± 0.05 kg (0.12, 0.30) DED 2 β 0.16 ± 0.06 kg FTO β 0.68 ± 0.25 (0.44, 0.93)	Each 1 kJ/g DED at age 10 years was positively associated with fat mass at age 13 years (*p* 0.05)
Kring and Heitman (2008) [35]	Continuous	Multivariate models, multiple linear regression model	TEEDLWEI/TEEDLW (doubly labeled water) EI/EE Defined as reporting bias ratio.	Z-score, age, physical activity level, dietary volume and puberty at baseline.	Normal weight 4.6 Overweight 4.4	BMI Z-score Boys Crude β –0.02 CI (–0.25; 0.15) *p* 0.60Adjusted β–0.04 CI (–0.29; 0.20) *p* 0.88 BMI Z-score Girls Crude β 0.21 CI (–0.3; 0.31) *p* 0.93 Adjusted β 0.23 CI (–0.07; 0.53) *p* 0.51	No significant association between DED and subsequent excess weight change was seen. DED was not associated with weight gain among children going through puberty
McCaffrey et al. (2008) [32]	Continuous and categorical (DED calculation method)	Logistic regression model adjusted for covariables	EI:EE Used as a covariate in the models	Sex, pubertal status, EI:EE, ED method	ED All 5.20 (4.93–5.92) ED Food: 8.28 (7.53–8.85) ED Soup:8.22 (7.53–8.81) ED solid: 9.17 (8.54–9.97) ED energy: 6.07 (5.59–6.49)	ED all OR 1.2 CI (0.53, 2.9) *p* 0.629 ED Food OR 2.1 CI (1.08, 4.17) *p* 0.029 ED soup OR 2.2 CI (1.09, 4.25) *p* 0.026 ED solid OR 1.9 CI (1.05, 3.57) *p* 0.033 ED energy OR 1.6 CI (0.65, 3.90) *p* 0.306	It depends on the method of calculation: association with FMI, but not with change in %BF, BMI, z-scores or WC. No association was found when beverages were included
Murakami et al. (2012) [38]	Categorical: ED categorized at quintile points	Logistic regression model crude and adjusted for covariables	EI:EER Used as a covariate in the models	Age, paternal and maternal educational level, television or computer game use, municipality, habitual exercise rate of eating,EI:EER, dietary glicemic load and energy intake from beverages	Adolescents 5.1	DED Quintile adjusted OR Boys Q2 0.98 CI (0.73,1.33) Q3 0.85 CI (0.63,1.16) Q4 0.90 CI (0.66,1.22) Q5 0.78 CI (0.57, 1.07) p 0.10 Girls Q2 0.85 CI (0.62,1.18) Q3 0.60 CI (0.42,0.85) Q4 0.65 CI(0.46,0.92) Q5 0.86 CI (0.61–1.20) p 0.12	DED was not associated with BMI in adolescents.
O’Sullivan et al. (2015) [40]	Continuous	Multivariate adjusted models, logistic regression and multiple linear regression	EI:EER Individuals were categorized as plausible, underreporters and overreporters. It was included as a categorical covariate in all analyses	Model 1: Adjusted for sex, family income, maternal education, puberty stage and physical, activity/screen use. Model 2: Model 1 adjusted for total daily kJ intake. Misreporting	4.46	BMI Foods and beverages Model 1 0.83 (0.70, 0.99) *p* 0.04 Model 2 0.85 (0.71, 1.01) *p* 0.07 Foods Model 1. 0.87 (0.77, 0.99) *p* 0.04 Model 2. 0.90 (0.80, 1.02) *p* 0.08 Waist-height ratio Foods and beverages 1. 0.86 (0.74, 1.01) *p* 0.06 2. 0.88 (0.75, 1.03) *p* 0.12 Foods 1. 0.88 (0.79, 0.99) *p* 0.03 2. 0.90 (0.80, 1.01) *p* 0.08	ED measures and dairy intake were inversely associated with obesity after adjustment for confounders; associations became non-significant after energy adjustment.
Schröder et al. (2013) [41]	Continuous and categorical: quartiles of DED	Multiple linear regression analysis, multiple logistic regression models	EI:BMR	Model 1: Sex and age Model 2: Sex, age., Leisure-time physical activity, low energy reporting, dietary fiber, maternal educational level, population size and energy intake from beverages	Kcal/g Q1: 0.94 Q2: 1.30 Q3: 1.57 Q4: 2.09	1. β −0.001 (−0.003, 0.001) *p* 0.005 2. β 0.003 (0.001,0.005) *p* 0.004 WC residuals 0.724 (0.377; 1.076) *p* <0.001	Higher DED is a risk for increased central fat distribution. DED was positively associated with abdominal obesity
Van Sluijs et al. (2016) [28]	Continuous	Multiple linear regression analysis and multiple logistic regression model	EI:EER Underreporting included as a continuous variable in the models	Model 1: Age, Sex Model 2. Model 1 socio-economic status, birth weight, maternal BMI, puberty status at follow-up, sleep duration. Model 3: model 2 + baseline DED for PA exposures and baseline MVPA for DED. Energy intake (kJ) from drinks and under-reporting.	At baseline 7.7 At follow-up 0.45	DED at baseline: WC: β 0.72 (0.26,1.17) FMI: β 0·22 (−0·08, 0·52) % BF: β 0·18 (−0·14, 0·50) DED at follow-up: WC: β −0·27 (−1·02, 0·48) FMI:β −0.86 (−1·59, −0·12) % BF:β −0.86 (−1·25, −0·11)	Positive association between DED and WC at baseline but not at follow-up. No association with FMI and %BF at baseline or follow up. The directions of associations with DED were inconsistent.
Zhou et al. (2015) [39]	Categorical: tertiles of ED (T1–T3)	Multivariate regression models (Linear trends) ^2^	EI:EER Underreporters were excluded from the analysis	Age, birth weight; exclusive breastfeeding duration; the timing of adding complementary foods; physical activity; parental education level; overweight parental BMI, smoking in the house; the percentage of EI from protein, fat, carbohydrate, and fiber intake	ED 1. 4.1 ED2. 6.75 ED3. 5.8 ED4. 6.5 ED 5. 5.6	DED Tertiles BMI z-score Boys Tertil 1. 0.2 CI (0.1, 0.4) Tertil 2. 0.2 CI (0.1, 0.4) Tertil 3. 0.1 CI (0.1, 0.3) *p* 0.9 Girls Tertil 1. 0.3 CI (0.1, 0.5) Tertil 2. 0.4 CI (0.1, 0.6) Tertil 3. 0.5 CI (0.3, 0.7) *p* 0.3 FMI Boys Tertil 1. 3.3 CI(2.9, 3.7) Tertil 2. 3.4 CI (3.1, 3.8) Tertil 3. 3.5 CI (3.1, 3.8) *p* 0.2 Girls Tertil 1 3.2 CI (2.9, 3.5) Tertil 2 3.2 CI (2.9, 3.5) Tertil 3 3.3 CI (3.0, 3.6) *p* 0.9	No association was found between DED and BMI, FMI, FFMI, WHR and %BF.

^1^ Recommended DED: <5.3 kJ/g [43]; energy—dense foods defined as those with an energy content of more than about 9.4–11.5 kJ/g [43]; ^2^ Linear trends:(*p* for trend) were tested with mean daily ED of all solid foods and drinks as continuous variables; Abbreviations: ATO: age at take-off; ALSPAC: Avon longitudinal Study of Parents and Children; BDHQCA: Diet history questionnaire for Japanese children and adolescents; %BF: body fat percentage ; BMI: Body mass index BMR: Basal metabolic rate; DED Dietary energy density; DLW: Double labeled water; DP: Dietary pattern; DONALD: Dortmund Nutritional Anthropometric Longitudinally Designed Study; ED: energy density or dietary energy density; EER: Estimated energy requirement EE: Energy expenditure; EI: Energy intake; FFMI: Fat free mass index; FMI:Fat mass index; FFQ: food-frequency questionnaire; Lesmean: Least square mean; MVPA: moderate-to-vigorous physical activity. NR: Not reported; OW/O: Overweight or obesity; RYUCHS: Ryukyus Child Health Study; PA Physical activity; SDS: Standard deviation score; SPEEDY: Sport, Physical activity and Eating behavior: Environmental Determinants in Young people; TEE: Total energy expenditure WC: Waist circumference; WHR: waist-to-hip ratio; WR Waist height ratio.

## References

[1] Das J.K., Salam R.A., Thomburg K.L., Prentice A.M., Campisi S., Lassi Z.S., Koletzko B., Bhutta Z.A. (2017). Nutrition in adolescents: Physiology, metabolism, and nutritional needs. Ann. N. Y. Acad..

[2] Birch L., Savage J.S., Ventura A. (2007). Influences on the development of children’s eating behaviours: From infancy to adolescence. Can. J. Diet. Pract. Res..

[3] Livingstone M.B., Robson P.J., Wallace J.M. (2004). Issues in dietary intake assessment of children and adolescents. Br. J. Nutr..

[4] Livingstone M.B., Robson P.J. (2000). Measurement of dietary intake in children. Proc. Nutr. Soc..

[5] Sabin M., Kiess W. (2015). Childhood obesity: Current and novel approaches. Best Pract. Res. Clin. Endocrinol. Metab..

[6] Rivera J.A., de Cossio T.G., Pedraza L.S., Aburto T.C., Sánchez T.G., Martorell R. (2014). Childhood and adolescent overweight and obesity in Latin America: A systematic review. Lancet Diabetes Endocrinol..

[7] Ng M., Fleming T., Robinson M., Thomson B., Graetz N., Margono C., Mullany E.C., Biryukov S., Abbafati C., Abera S.F. (2013). Global, regional, and national prevalence of overweight and obesity in children and adults during 1980–2013: A systematic analysis for the Global Burden of Disease Study. Lancet.

[8] Spruijt-Metz D. (2011). Etiology, treatment and prevention of obesity in childhood and adolescence: A decade in review. J. Res. Adolesc..

[9] Buckland G., Bach A., Serra-Majem L. (2008). Obesity and the Mediterranean diet: A systematic review of observational and intervention studies. Obes. Rev..

[10] Pérez-Escamilla R., Obbagy J.E., Altman J.M., Essery E.V., McGrane M.M., Wong Y.P., Spahn J.M., Williams C.L. (2012). Dietary energy density and body weight in adults and children: A systematic review. J. Acad. Nutr. Diet..

[11] Rouhani M., Haghighatdoost F., Surkan P., Azadbakht L. (2016). Associations between dietary energy density and obesity: A systematic review and meta-analysis of observational studies. Nutrition.

[12] O’Connor L., Walton J., Flynn A. (2013). Dietary energy density and its association with the nutritional quality of the diet of children and teenagers. J. Nutr. Sci..

[13] Patterson E., Wärnberg E., Poortvliet E., Kearny J.M., Sjöström M. (2010). Dietary energy density as a marker of dietary quality in Swedish children and adolescents: The European Youth Heart Study. EJCN.

[14] Drewnowski A., Almiron-Roig E., Marmonier C., Lluch A. (2004). Dietary energy density and body weight: Is there a relationship?. Nutr. Rev..

[15] Rolls B.J. (2009). The relationship between dietary energy density and energy intake. Physiol. Behav..

[16] Johnson L., Wilks D.C., Lindroos A.K., Jebb S.A. (2009). Reflections from a systematic review of dietary energy density and weight gain: Is the inclusion of drinks valid?. Obes. Rev..

[17] Costa C., Del-Ponte B., Assunção M., Santos I. (2018). Consumption of ultra-processed foods and body fat during childhood and adolescence: A systematic review. Public Health Nutr..

[18] Ledikwe J.H., Blanck H.M., Kette L., Serdula M.K., Seymour J.D., Tohill B.C., Rolls B.J. (2006). Dietary energy density is associated with energy intake and weight status in US adults. Am. J. Clin. Nutr..

[19] Mendoza J.A., Drewnowski A., Christakis D.A. (2007). Dietary energy density is associated with obesity and the metabolic syndrome in US adults. Diabetes Care.

[20] Johnson L., Mander A.P., Jones L.R., Emmett P.M., Jebb S.A. (2008). Energy-dense, low-fiber, high-fat dietary pattern is associated with increased fatness in childhood. Am. J. Clin. Nutr..

[21] Wilks D.C., Mander A.P., Jebb S.A., Thompson S.G., Sharp S.J., Turner R.M., Lindroos A.K. (2011). Dietary energy density and adiposity: Employing bias adjustments in a meta-analysis of prospective studies. BMC Public Health.

[22] Birch L.L., Deysher M. (1986). Caloric compensation and sensory specific satiety: Evidence for self-regulation of food intake by young children. Appetite.

[23] Leahy K.E., Birch L.L., Rolls B.J. (2008). Reducing the energy density of multiple meals decreases the energy intake of preschool-age children. Am. J. Clin. Nutr..

[24] Cecil J.E., Palmer C.N., Wrieden W., Murrie I., Bolton-Smith C., Watt P., Wallis D.J., Hetherington M.M. (2005). Energy intakes of children after preloads: Adjustment, not compensation. Am. J. Clin. Nutr..

[25] Fisher J.O., Liu Y., Birch L.L., Rolls B.J. (2007). Effects of portion size and energy density on young children’s intake at a meal. Am. J. Clin. Nutr..

[26] Johnson L., Mander A.P., Jones L.R., Emmett P.M., Jebb S.A. (2008). A prospective analysis of dietary energy density at age 5 and 7 years and fatness at 9 years among UK children. Int. J. Obes..

[27] Aburto T., Cantoral A., Hernández L., Alicia L., Carriquiry A.L., Rivera J. (2015). Usual dietary energy density distribution is positively associated with excess body weight in Mexican children. J. Nutr..

[28] Van Sluijs E.M., Sharp S.J., Ambrosini G.L., Cassidy A., Griffin S.J., Ekelund U. (2016). The independent prospective associations of activity intensity and dietary energy density with adiposity in young adolescents. Br. J. Nutr..

[29] Moher D., Liberati A., Tetzlaff J., Altman D.G. (2009). The PRISMA Group (2009) Preferred Reporting Items for Systematic Reviews and Meta-Analyses: The PRISMA Statement. PLoS Med..

[30] Wells G.A., Shea B., O’Connell D., Peterson J., Welch V., Losos M., Tugwell P. (2014). The Newcastle-Ottawa Scale (NOS) for Assessing the Quality of Non-Randomised Studies in Meta-Analyses.

[31] Alexy U., Sichert-Hellert W., Kersting M., Schultze-Pawlitschko V. (2004). Pattern of long-term fat intake and BMI during childhood and adolescence results of the DONALD Study. Int. J. Obes. Relat. Metab. Disord..

[32] McCaffrey T.A., Rennie K.L., Kerr M.A., Wallace J.M., Hannon-Fletcher M.P., Coward W.A., Jebb S.A., Livingstone M.B. (2008). Energy density of the diet and change in body fatness from childhood to adolescence: Is there a relation?. Am. J. Clin. Nutr..

[33] Gunther A., Stahl L., Buyken A., Kroke A. (2012). Association of dietary energy density in childhood with age and body fatness at the onset of the pubertal growth spurt. Am. J. Clin. Nutr..

[34] Ambrosini G.L., Emmett P.M., Northstone K., Howe L.D., Tilling K., Jebb S.A. (2012). Identification of a dietary pattern prospectively associated with increased adiposity during childhood and adolescence. Int. J. Obes..

[35] Kring S.I., Heitmann B.L. (2008). Fiber intake, not dietary energy density, is associated with subsequent change in BMI z-score among sub-groups of children. Obes. Facts.

[36] Johnson L., van Jaarsveld C.H., Emmett P.M., Rogers I.S., Ness A.R., Hattersley A.T., Timpson N.J., Smith G.D., Jebb S.A. (2009). Dietary energy density affects fat mass in early adolescence and is not modified by FTO variants. PLoS ONE.

[37] Butte N.F., Cai G., Cole S.A., Wilson T.A., Fisher J.O., Zakeri I.F., Ellis K.J., Comuzzie A.G. (2007). Metabolic and behavioral predictors of weight gain in Hispanic children: The Viva la Familia Study. Am. J. Clin. Nutr..

[38] Murakami K., Miyake Y., Sasaki S., Tanaka K., Arakawa M. (2012). An energy-dense diet is cross-sectionally associated with an increased risk of overweight in male children, but not in female children, male adolescents, or female adolescents in Japan: The Ryukyus child health study. Nutr. Res..

[39] Zhou X., Xue H., Duan R., Liu Y., Zhang L., Harvey L., Cheng G. (2015). The Cross-sectional association of energy intake and dietary energy density with body composition of children in Southwest China. Nutrients.

[40] O’Sullivan T., Bremner A.P., Bremer H.K., Seares M.E., Beilin L.J., Mori T.A., Lyons-Wall P., Devine A., Oddy W.H. (2015). Dairy product consumption, dietary nutrient and energy density and associations with obesity in Australian adolescents. J. Hum. Nutr. Diet..

[41] Schröder H., Méndez M.A., Gómez S.F., Fito M., Ribas L., Aranceta J., Serra-Majem L. (2013). Energy density, diet quality, and central body fat in a nationwide survey of young Spaniards. Nutrition.

[42] Forrestal S.G. (2011). Energy intake misreporting among children and adolescents: A literature review. Matern. Child Nutr..

[43] WCRF/AICR (2007). Food, Nutrition, Physical Activity, and the Prevention of Cancer: A Global Perspective.

[44] Vernarelli J., Mitchell D.C., Hartman T., Rolls B. (2011). Dietary energy density is associated with body weight status and vegetable intake in U.S. Child. J. Nutr..

[45] Murakami K., Sasaki S., Takahashi Y., Uenishi K., Japan Dietetic Students’ Study for Nutrition and Biomarkers Group (2007). Dietary energy density is associated with body mass index and waist circumference, but not with other metabolic risk factors, in free-living young Japanese women. Nutrition.

[46] Hartline H., Rose D., Johnson C., Rice J., Webber L. (2009). Energy density of foods, but not beverages, is positively associated with body mass index in adult women. Eur. J. Clin. Nutr..

[47] Cox D.N., Mela D.J. (2000). Determination of energy density of freely selected diets: Methodological issues and implications. Int. J. Obes..

[48] Peterson C.M., Su H., Thomas D.M., Heo M., Golnabi AH., Pietrobelli A., Heymsfield S.B. (2017). Tri-Ponderal Mass Index vs. Body Mass Index in Estimating Body Fat During Adolescence. JAMA Pediatr..

[49] Livingstone M.B., Black A.E. (2003). Markers of the validity of reported energy intake. J. Nutr..

[50] Burrows T., Martin R., Collins A. (2010). Systematic review of the validity of dietary assessment methods in Children when compared with the method of doubly labeled Water. J. Am. Diet. Assoc..

[51] Collins C., Watson J., Burrows T. (2010). Measuring dietary intake in children and adolescence in the context of overweight and obesity. Int. J. Obes..

[52] Arango-Angarita A., Shamah-Levy T., Rodríguez-Ramírez S. (2018). Dietary energy density is associated with body mass index-for-age in Mexican adolescents. Matern. Child Nutr..

[53] Méndez M., Popkin B., Buckland G., Schroder H., Amiano P., Barricarte A., Huerta J.M., Quirós J.R., Sánchez M.J., González C.A. (2011). Alternative methods of accounting for underreporting and overreporting when measuring dietary intake-obesity relations. Am. J. Epidemiol..

[54] Rennie K., Coward A., Jebbi S. (2007). Estimating under-reporting of energy intake in dietary surveys using an individualized method. Br. J. Nutr..

[55] Mendoza J.A., Drewnowski A., Cheadle A., Christakis D.A. (2006). Dietary energy density is associated with selected predictors of obesity in U.S. children. J. Nutr..

